# Identification of Genes Affecting Vacuole Membrane Fragmentation in *Saccharomyces cerevisiae*

**DOI:** 10.1371/journal.pone.0054160

**Published:** 2013-02-01

**Authors:** Lydie Michaillat, Andreas Mayer

**Affiliations:** Département de Biochimie, Université de Lausanne, Epalinges, Switzerland; Université de Nice-CNRS, France

## Abstract

The equilibrium of membrane fusion and fission influences the volume and copy number of organelles. Fusion of yeast vacuoles has been well characterized but their fission and the mechanisms determining vacuole size and abundance remain poorly understood. We therefore attempted to systematically characterize factors necessary for vacuole fission. Here, we present results of an *in vivo* screening for deficiencies in vacuolar fragmentation activity of an ordered collection deletion mutants, representing 4881 non-essential genes of the yeast *Saccharomyces cerevisiae*. The screen identified 133 mutants with strong defects in vacuole fragmentation. These comprise numerous known fragmentation factors, such as the Fab1p complex, Tor1p, Sit4p and the V-ATPase, thus validating the approach. The screen identified many novel factors promoting vacuole fragmentation. Among those are 22 open reading frames of unknown function and three conspicuous clusters of proteins with known function. The clusters concern the ESCRT machinery, adaptins, and lipases, which influence the production of diacylglycerol and phosphatidic acid. A common feature of these factors of known function is their capacity to change membrane curvature, suggesting that they might promote vacuole fragmentation via this property.

## Introduction

Many organelles adjust their morphology by fusion or fragmentation. Size, copy number and shape can change in reproducible ways in response to changes in environmental conditions or during the cell cycle. Examples for organelles undergoing regulated fusion and fission include endosomes, lysosomes, peroxisomes, the Golgi, mitochondria and chloroplasts [1]–[8]. Organelle fragmentation may serve different purposes, depending on the organelle. For low-copy organelles it can facilitate their transmission to daughter cells [9]. The Golgi matrix, for example, divides, attaches to the spindle and is thus distributed during mitosis. The membranes regenerating the stacks are partially derived from ER [10], [11]. For endosomes, a larger copy number of the organelle may be necessary to allow distribution of the organelle into different regions of the cell. Producing enough organelle copies to deposit them in different parts of the cell may help to regulate signaling, since the location of an endosome modifies the efficiency of signaling from endocytosed receptors residing in it [12], [13].

For vacuoles and lysosomes there might be additional reasons to undergo regulated cycles of fission and fusion. Vacuoles function in autophagy, osmoregulation and storage of amino acids and ions [14]. Regulation of lysosomal/vacuolar hydrolytic capacity is crucial to the correct functioning of eukaryotic cells, as shown by the fact that mutations in genes affecting lysosomal degradation give rise to numerous lysosomal storage diseases [15]. An increase in vacuolar hydrolytic capacity by enhanced expression of vacuolar hydrolases is paralleled by significant changes in vacuolar structure. During logarithmic growth on rich media, a yeast cell contains 2–5 vacuoles of intermediate size. Upon nutrient limitation and induction of autophagy, they coalesce into a single organelle and thus expand their volume, facilitating the degradation of cytoplasmic material that is transferred into vacuoles under these conditions [16]–[18]. Similarly, the lysosomal compartment of mammalian cells increases in size upon induction of autophagy. Also osmotic changes affect vacuole structure. Hypotonic media promote vacuole coalescence whereas hypertonic conditions induce rapid fragmentation [19]. Vacuoles may respond in this way because fragmentation and coalescence change their surface to volume ratio. Under hypertonic conditions, cells loose water. The vacuole volume is reduced but the membrane surface remains constant. Fragmentation of the organelle can then readjust the surface to the reduced volume under these conditions.

It is reasonable to assume that transitions in organelle size and number are the product of a regulated equilibrium between the fundamental processes of organelle membrane fission and fusion. The mechanism of membrane fusion on organelles has been extensively studied. Similarly, satisfying hypotheses on membrane fission in the biogenesis of coated transport vesicles have been obtained [20], [21]. But fission of entire organelles and its coordination with the antagonistic fusion activities remains poorly understood. Several proteins needed for organelle fragmentation have been identified, particularly for mitochondria and the Golgi [12], [22], but we are still lacking a coherent understanding of the fragmentation mechanism. It is not clear whether the machineries for fission of different organelles are related or whether fission occurs in unique, organelle-specific ways. One element hinting at common mechanisms is the fact that dynamin-like GTPases are important for fission of several organelles, such as mitochondria, chloroplasts and vacuoles [1], [3]–[6], [8], [11], [21]. Dynamin-like GTPases can form large ring-like assemblies that can surround fission sites and they might act as mechano-chemical constriction devices during fission [23]–[25]. Dynamins are also abundant at sites of actin remodeling and actin-driven vesicle motility, suggesting that they participate in active transport of vesicles along cytoskeletal tracks and in actin reorganization [26].

Yeast vacuoles are good models for organelle transmission, organelle fragmentation and membrane fusion [19]. They undergo regulated cycles of membrane fission and fusion. Vacuoles can be isolated in good yield and purity and their fragmentation could recently be reconstituted in a cell-free system [27]. Since yeast vacuoles are large organelles with a usual diameter of 1 to 3 µm, their shape, size and number can be analyzed by fluorescence microscopy, for example after staining their membrane with the lipophilic dye FM4-64 [28]. During the cell cycle or in response to hypertonic shock [29] vacuoles fragment into numerous smaller vacuoles with a diameter below 0.5 µm. Microscopic analysis of vacuole structure permitted the identification of several factors involved in vacuole fragmentation activity: the PI(3)P-5 kinase Fab1p, its cofactors Vac14p, Vac7p and Fig4p, and the PI(3,5)P_2_ binding protein Atg18p, all of which are required to maintain the vacuolar lipid PI(3,5)P_2_ [30]–[39]. Vacuole fragmentation requires the electrochemical potential over the vacuole membrane that is established by the V-ATPase [40]. Furthermore, it requires the CORVET complex subunit Vps3p [41], TOR kinase [27] and Yck3p, a casein kinase that counteracts rapid refusion of the vacuole after fragmentation [42]. Also a dynamin-like GTPase, Vps1p, acts in vacuole fission in S. cerevisiae [43]. The membrane association of Vps1p is influenced by the SNARE-activating ATPase Sec18p/NSF and the t-SNARE Vam3p.

Here, we describe a systematic approach to identify factors required for vacuole fragmentation. We screened the mutants for all non-essential yeast genes for the inability to fragment vacuoles in response to salt addition [30].

## Results and Discussion

Vacuoles fragment upon addition of salt to the media. Since this reaction occurs synchronously and in less than 10 minutes it offers the possibility to screen the yeast knockout collection, which contains 4881 mutants, each deleted for one defined non-essential ORF [44]. Using this collection obviates the necessity of mapping and cloning a screened mutant, which would be difficult after a random mutagenesis approach. Screening the collection for mutants defective in vacuole fragmentation posed several challenges. Some mutant strains have highly fragmented vacuoles on standard rich media [45], precluding analysis of their fragmentation by light microscopy. Vacuole structure is also influenced by the type of medium used, by nutrient availability and by growth phase. Starvation, for example, inactivates TORC1, which is necessary for vacuole fragmentation [27]. Nutrient limitation hence interferes with the detection of vacuole fragmentation and has to be avoided during growth of the cells. Finally, vacuole fragmentation upon osmotic shock is a transient phenomenon. It can be observed 10–20 min after salt shock, depending on the severity of osmotic change [30], [42]. Later, vacuoles regain their normal shape and number as the cells adapt to the higher osmotic values by a variety of long-term adaptive mechanisms [46]. Thus, care has to be taken concerning the growth conditions and the timing of the experiment.

**Figure 1:**
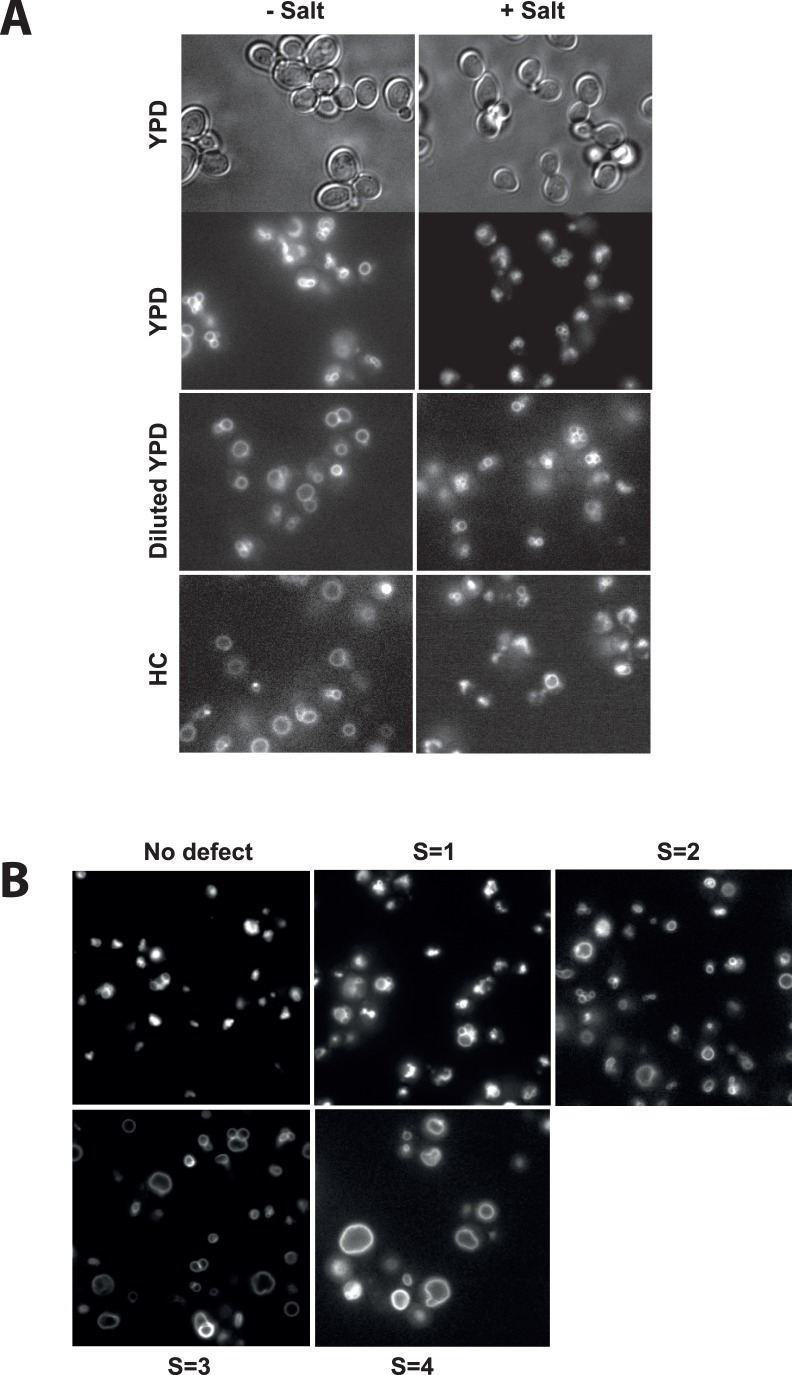
The in vivo fragmentation assay under screening conditions. (A) The cells were grown overnight in 96-well plates in HC-Leu^-^ medium to an OD_600_<2. They were diluted 10-fold in YPD, stained with 20 µM FM4-64 for 1 hour, centrifuged and resuspended in YPD, HC or YPD diluted 5-fold with water (diluted YPD). After shaking for 2 hrs at 27°C, cells were transferred into optical 96-well plates. Fragmentation was induced by supplementing the suspension with 0.4 M NaCl. After 10 min of incubation at room temperature, cells were analyzed by fluorescence microscopy. Note that the screen was performed on a non-confocal microscope. Fragmentation was easier to judge on the microscope than on the photos, due to the possibility to focus through the sample in the z-direction. (B) Examples illustrating the scoring of the fragmentation defect. Samples sho pictures from cells after incubation with salt as in A.

In order to grow the cells reproducibly we precultured them at 30°C in 96-wells plates in HC-leu^-^ medium. Overnight cultures were inoculated from these plates and incubated (15 h, 25°C, 150 rpm) such that they were still in logarithmic phase (OD_600_<1) the next morning. In the morning, the cultures were diluted up to 10-fold into YPD containing 20 µM of FM4-64, a vital dye staining the vacuolar boundary membrane (Vida & Emr, 1995). The cells were shaken for another hour at 27°C in order to permit uptake of the dye into the cells. The plates were centrifuged and the supernatant was exchanged for fresh medium without FM4-64. The cells were then cultivated further for 2 hours at 27°C in order to allow redistribution of FM4-64 into vacuolar membranes, where this dye finally accumulates. Then, the cells were transferred into optical 96-well plates with a glass bottom of 0.17 mm thickness for microscopic analysis. The entire bottom was covered with immersion oil so that the entire 96-well plate could directly be analyzed with a 100× 1.4 NA lens on an inverted fluorescence microscope. This permits to proceed from one well to the next within 10–15 seconds. Each plate was scored twice, once before the addition of salt and once 10 min after the addition of 0.4 M NaCl, a treatment that induces vacuole fragmentation (Fig. 1). Since salt-induced vacuole fragmentation is a transient phenomenon, the addition of salt was not performed for the entire plate at the same time. It was phased so as to guarantee that the incubation time with salt was close to 10 min for the wells of each row.

Direct visualization in 96-well plates allowed a single person to screen about 300 mutants per afternoon. A problem that became apparent during the first round of screening was due to the background of the knockout collection. The collection had been created in BY4741 cells. In comparison to many other common laboratory strains these cells are relatively small and carry 3–6 small vacuoles during logarithmic growth in YPD. Fragmentation of these vacuoles is still detectable upon salt treatment but it requires careful inspection (Fig. 1A). In order to improve the morphological situation, we therefore modified the conditions for a subsequent round of screening by resuspending the cells in YPD diluted 5- fold with water after the FM4-64 staining. This change leads to a partial fusion of vacuoles, resulting in fewer and bigger vacuoles whose morphology is easier to evaluate (Fig. 1A). This method thus allows recognizing the phenotype more easily and to screen faster. Fragmentation in diluted YPD produces even smaller and more numerous vacuoles than in normal YPD. Another means of increasing vacuolar size and decreasing vacuole number in BY4741 cells is to grow them on Hartwell’s complete (HC) medium (Fig. 1A). Therefore, we performed another round of screening in HC complete medium. All mutants were thus screened in normal YPD, in diluted YPD and in HC medium. A potential advantage of screening the collection under three different conditions is that the results can help to distinguish core elements necessary for vacuole fragmentation from mutations affecting regulatory factors specific to the particular growth condition. Core processes necessary for fragmentation should be identified independently of the condition, whereas mutations affecting adaption to environmental conditions might show effects only on one specific medium.

In order to rank the candidates we attributed a score to them that reflects the estimated degree of their loss of fragmentation activity; S = 1 if 5–10% of cells showed non-fragmented vacuoles after salt treatment; S = 2 for 20–50% of cells with non-fragmented vacuoles; S = 3 for 60–90%; and S = 4 if all cells showed non-fragmented vacuoles. The scores from the three screening approaches were summed up. They had to be normalized since mutants that did not grow or survive under one or the other condition were scored less often. In order to account for this, the sum of the scores was divided by the number of successful analyses each mutant underwent, yielding a phenotypic score P with a scale from 0 to 4. We defined 0<P<1.3 as small defect, 1.3≤P<1.6 as a moderate defect, 1.6≤P<2 as a strong defect and 2≤P≤4 as a very strong defect (Fig. 1B). The P scores obtained were in the range of 0 in the case of no inhibition of fragmentation to 3.5 for the strongest mutants.

From the 4881 strains in the knockout collection, small defects in fragmentation activity were observed for 2653 mutants, moderate defects for 150 mutants, strong defects for 70, very strong defects for 63. This suggests that the scoring criteria for the small defects were too subtle to be useful. We focused on mutants with strong and very strong phenotypes (Tab.1). Of the 133 mutants with a strong defect of fragmentation activity 83% (110) concerned known ORFs and 17% (22) concerned uncharacterized ORFs (Table 1). For comparison, of the 4881 strains in the knockout collection 12% (685) concern uncharacterized ORFs. Thus, there is a small enrichment of uncharacterized ORFs among the strong mutants recovered in the screen.

**Table 1:** Open reading frames of unknown function.

ORF	Gene name(s)	Score
YKL061W	BLI1	3.0
YLR358C		3.0
YNL324W		2.8
YPR116W	RRG8	2.5
YDR509W		2.3
YJR061W		2.3
YDR215C		2.0
YLR169W		2.0
YOR302W		2.0
YDL151C	BUD30	2.0
YEL072W	RMD6	2.0
YML002W		1.8
YMR003W	AIM34	1.8
YCR102W-A		1.8
YGR160W		1.8
YHR151C	MTC6	1.8
YLR415C		1.8
YLR422W		1.8
YNL228W		1.8
YLR235C		1.7
YOR024W		1.7
YPR099C		1.7

### Identification of known Factors of Vacuole Fragmentation by the Screen

**Figure 2:**
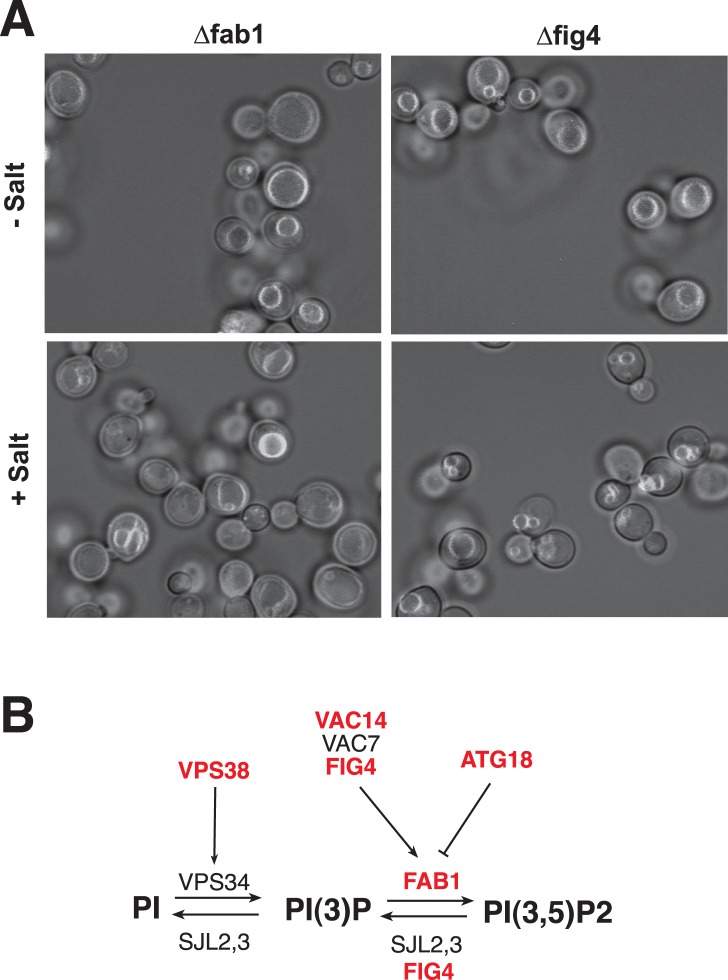
Phenotype of mutants in the PI(3,5)P_2_ pathway under screening conditions. (A) BY4741 *Δfab1* and BY4741 *Δfig4* cells were cultivated and subjected to vacuole fragmentation under screening conditions in diluted YPD. Pictures show an overlay of the fluorescence and brightfield channels. (B) Metabolic pathways leading to the synthesis of PI(3,5)P_2_. Steps for which a gene deletion led to strong or moderate deficiency in vacuole fragmentation are indicated in red/bold.

Factors already known to be involved in vacuole fragmentation were re-identified in the screen: proteins that regulate the levels of phosphatidylinostol-3,5-bisphosphate (PI(3,5)P_2_) and the V-ATPase. The identification of numerous genes known to be required for vacuole fragmentation validates the screening method. Among the PI(3,5)P_2_-related genes identified in the screen is Fab1p, a phosphatidylinositol-3-phosphate-5-kinase (Fig. 2; Table 2). Vac14p and Fig4p are Fab1p-associated factors that regulate PI(3,5)P_2_ levels both positively and negatively, resulting in tight control and pronounced changes of PI(3,5)P_2_ upon osmotic challenge [30], [33], [36]–[39], [47]–[49]. Vps38p is a subunit of the PI-3-kinase complex II that produces PI(3)P, the substrate for PI(3,5)P_2_ synthesis [50]. Atg18p regulates PI(3,5)P_2_ levels negatively. It is a PI(3,5)P_2_ binding protein and putative effector of this lipid that is also involved in vacuole fragmentation [32], [34], [35], [51]. We also recovered the Vma2p and Vma6p subunits of the V-ATPase, the proton pump that acidifies the vacuolar lumen. This reflects the fact that the electrochemical potential across the vacuolar membrane is necessary for vacuole fragmentation [40]. Vacuole fragmentation also requires the TORC1 complex and Sit4p, a PP2A-like phosphatase acting downstream of TORC1 [27]. The screen identified moderate fragmentation defects for Δ*tor1* and Δ*sit4* cells (Tab. 2). Taken together, the re-identification of numerous genes known to be involved in vacuole fragmentation validates the screening method.

**Table 2:** Identified mutants affecting processes known to be involved in vacuole fragmentation: PI(3,5)P_2_ metabolism, vacuole acidification and TOR signaling.

ORF	Gene name(s)	Function	Score
YFR019W	FAB1	PI(3)P kinase, vacuolar sorting and vacuole homeostasis	2.3
YNL325C	FIG4	PI(3,5)P_2_ phosphatase, response to osmotic shock, regulation of vacuole size	2.8
YLR386W	VAC14	Regulates synthesis of PI(3,5)P_2,_ vacuolar sorting, vacuole homeostasis	2.0
YFR021W	ATG18	Binds PI(3,5)P_2_ and regulates FAB1 activity	2.5
YLR360W	VPS38	Subunit of PI-3-kinase complex II	2.3
YKL135C	APL2	β-subunit of AP-1 complex; mutation reduces PI(3,5)P_2_	3.0
YBR127C	VMA2	Subunit B of the V-ATPase	2.0
YLR447C	VMA6	Subunit d of the V-ATPase	1.3
YJR066W	TOR1	Subunit of TORC1	1.5
YDL047W	SIT4	Ser/Thr phosphatase	1.3

Like all screening procedures the approach performed here is subjected to limitations. One concerns the source material. The collection of deletion strains contains errors and individual strains may change over time, due to second site mutations [52]. Others concern our assays. We could not measure fission rates directly but rather scored the steady state morphology of vacuoles. The equilibrium between the vacuole fusion and fission, rather than their absolute rates, determines the observable vacuole morphology [40]. Therefore, genes identified in the screen may not only comprise membrane fission factors but also genes down-regulating vacuole fusion. Furthermore, some genes affect both fission and fusion of vacuoles. Whether mutation of a bi-functional gene will shift vacuole structure towards a fragmented or coalesced state then depends on its relative impact on the fusion and fission rates. This may lead to false negatives or positives. Contrary to our expectations, the mutant of the dynamin-like GTPase Vps1p scored only with a small defect. This is probably due to the fact that already in the absence of salt Δ*vps1* mutants in BY4741 background exhibit a complex vacuole structure with numerous smaller vacuoles that can only be resolved by confocal microscopy. This phenotype is likely due to the dual function of Vps1p in vacuole fusion and fission [43], which leads to varying vacuolar morphology depending on the strain background. Bi-functionality may also be the reason why the screen missed some subunits of the H^+^-pumping V-ATPase [53], which are necessary for vacuole fragmentation and fusion [54]. In addition, in order to grow V-ATPase mutants reproducibly, the medium should be buffered to acidic pH, which was not the case in our screen. For similar reasons we might have missed the PI-3-kinase Vps34p, which generates the precursor of PI(3,5)P_2_. *Δvps34* cells have proton pump defects and share many growth defects with V-ATPase mutants [55]. Moreover, Vps34p is also in the group of bi-functional factors, i.e. it is required also for vacuole fusion [56].

The screen missed the casein kinase Yck3p. Yck3p phosphorylates the t-SNARE Vam3p and inactivates the Vam2p subunit of the HOPS complex that is necessary for membrane tethering during vacuole fusion [42]. Δ*yck3* vacuoles do fragment upon salt addition but they cannot maintain this fragmented state. They re-fuse precociously due to the inability to maintain Vam2p phosphorylated and inactive [42]. *Δyck3* scored only with a mild deficiency (1.3) in salt-induced vacuole fragmentation (Table 3). This is likely due to the fact that we assayed the cells already 10 min after the salt shock, a time which is not sufficient to complete the accelerated re-fusion of vacuoles in Δ*yck3* cells.

**Table 3:** Mutants related to vacuolar function, biogenesis and inheritance.

ORF	Gene name(s)	Function	Score
**Vacuolar protein sorting**			
YOR036W	PEP12	t-SNARE in Golgi-to vacuole transport	1.7
YML097C	VPS9	GEF for Rab-GTPase Vps21	2.8
YOR089C	VPS21	Rab GTPase, vacuolar hydrolases sorting	1.5
YJR126C	VPS70	Vacuolar trafficking of Prc1p	1.5
YDR495C	VPS3	CORVET complex	1.7
YAL002W	VPS8	CORVET complex	2.0
YGR206W	MVB12	ESCRT-I subunit	3.0
YPL065W	VPS28	ESCRT-I-subunit	1.6
YPL002C	SNF8	ESCRT-II subunit, glucose de-repression	1.8
YLR417W	VPS36, VAC3	ESCRT-II subunit, vacuole inheritance	2.7
YLR025W	SNF7	ESCRT-III subunit	2.0
YPL084W	BRO1	Ubiquitin hydrolase in MVBs;	1.8
		SNF7 interactor	
YKR035W-A	DID2	ESCRT-III dissociation; Vps4 interactor	1.3
YLR181C	VTA1	Protein sorting at MVBs; Vps4 interactor	1.8
YDR486C	VPS60	Late endosome to vacuole transport; Vta1 interactor	1.8
**Vacuole fusion**			
YML001W	YPT7	Rab GTPase, vacuole fusion	1.6
YER123W	YCK3	Vacuole fusion during hypertonic stress	1.3
YLR396C	VPS33	SM-protein for vacuole fusion, HOPS-complex	1.8
**Other vacuole-related**			
YOR087W	YVC1	Vacuolar cation channel	1.5
YHR028C	DAP2	Di-peptidyl aminopeptidase	1.5
YJR001W	VMR1	Vacuolar ABC transporter	1.8
YDR128W	SEA3	SEA complex; associates with vacuoles	1.8

### Novel Candidates

The identified mutants with strong fragmentation deficiency can be classified into different functional families (Table 4); membrane traffic towards vacuoles, lipid modification, nucleus, enzymes, mitochondrial and ribosomal proteins and others. Mitochondrial and ribosomal mutants have often been selected in a large variety of genome-wide screens of the deletion mutant collection. This indicates a very pleiotropic phenotype of these mutants. The nuclear class of selected mutants affects DNA structure and gene expression. For these mutations, we suppose a rather indirect involvement in vacuole fragmentation because salt-induced fragmentation happens in 3–8 minutes, i.e. too fast for transcriptional and translational responses to play a direct role. Proteins involved in sporulation, such as Snf4p, emerged from the screen, together with proteins that have a common role in meiotic recombination, such as Rmd6p, Ndj1p and Mek1p. Several proteins are required for cell division and the cell cycle, such as Mbp1p, Mek1p and the cyclins Clb2p and Bur2p. The cyclin Clb2p is intriguing because fragmentation of the vacuolar membrane is also required for transmission of the organelle to daughter cells, a process coupled to the cell cycle (Wang et al, 1996). Clb2p affects the G2-M transition. Its deletion had a similar effect on vacuole fragmentation as deletion of the transcription factor Mbp1p, which, however, affects the G1 to S transition. These results can therefore not suggest a correlation of fragmentation competence with a specific phase of the cell cycle. This matches with our microscopic observations, which indicated that all cells in a population fragment their vacuoles under salt stress, irrespective of their cell cycle stage. Several of these proteins have been linked to vacuole-related phenotypes by high-throughput studies, for example vacuolar polyphosphate accumulation (Mek1p, Bur2p), osmo-tolerance (Clb2p) and heavy metal resistance (Bur2p).

**Table 4:** Identified mutants with strong defects.

Family of genes	Strong defect, score: [1.6–2]	Very strong defect, score: [2–3.5]
**TRAFFIC**		
**Endocytosis, cytoskeleton, vacuole fusion and fragmentation**	BRO1, YCK1, VTA1, SNF8, VPS60, VPS6, GGA2, VPS3,VPS28, GIC1, VPS33, YPT7, SEA3, JJJ1	MVB12, VPS9, VPS36, SNF7, VPS29, VMA2, PAC11,VPS55, VPS8
**Clathrin**	APL4, APS1, YAP1801	APL2, APS3
**Vacuolar ion homeostasis**	VMR1, PPZ1	
**LIPID MODIFICATION**	TGL5, PLB1, PLB2	FIG4, ATG18, FAB1, VAC14, VPS38, APQ12
**NUCLEUS**		
**Transcription and DNA structure**	PDR8, CDC73, DIA2, MOT3, CHL1, SPT10	RSC2, SPT7, NOT5, RAD7, RPA12, RPC53, HTL1,UME6, URE2
**Nuclear transport, RNA maturation**	HEK2, BRR1, DHH1	NUP133, APQ12
**Cell cycle, cell division and** **growth**	NDJ1, SNF4, MBP1, NSR1	CLB2, MEK1, BUR2, FPR1, EGO3, TPK2
**ENZYMES**	GTT1, BNA1, APA1	GAS5, ADH1, FYV9, GNP1, OSM1, RIB4, CPA1
**Affecting protein stability**	NTA1, UBR1, ULA1	UMP1
**MITOCHONDRIA**	DIC1, ISU2, MDM1, COX6, MPRS8, MIC17, MRH4, AEP3,RPO41	MSR1, MRPS35, MRPL17, MRP7, RSM22
**RIBOSOMES (cytosolic)**	RPS24A, RPS4A, RPS1A, RPS1B	RPS7A, RPL41B, RPS30A
**OTHER**	PEX15, PDR11, AHA1, SOL3	PEX28, YKE4
**ORFs of unknown function**	YML002W, YMR003W, YDL151C, YCR102W-A, YGR160W, YHR151C, YLR415C, YLR422W, YNL228W, YLR235C,YOR024W, YPR099C	YKL061W, YLR358C, YNL324W, YPR116W, YDR509W, YJR061W, YDR215C, YLR169W, YOR302W, YEL072W

Several of the identified mutants are related to TORC1 signaling, which is necessary for vacuole fragmentation [27]. Deletion of the peptidyl-prolyl isomerase gene FPR1 produced a very strong phenotype. Fpr1p binds to and inactivates TORC1 upon addition of rapamycin [57]. Aside from this pharmacologically induced interaction, however, no direct physiological links between Fpr1p and TORC1 have been identified so far. Deletion of the TORC1 activating protein Ego3p had a strong defect.

### Lipid Catabolic Pathways Influence Vacuole Fragmentation

**Figure 3:**
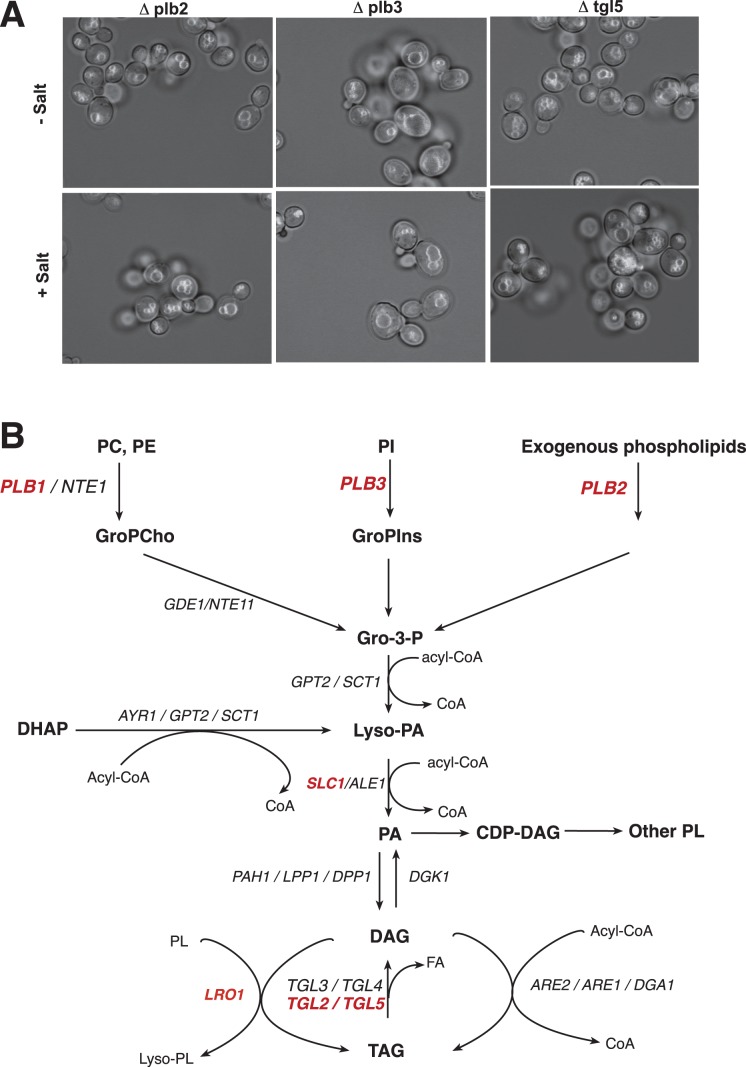
Mutants in pathways converging on dicacylglycerol and phosphatidic acid. (A) Phenotype of mutants related to triglyceride metabolism under screening conditions. Cells of the indicated mutants were grown and subjected to vacuole fragmentation as in Figure 1. Pictures show an overlay of the fluorescence and brightfield channels. (B) Scheme of pathways leading to the generation of diacylglycerol (DAG) and phosphatidic acid (PA). PC, phosphatidylcholine; PI, phosphatidylinositol; GroPCho, glycerophosphocholine; GroPIns, glycerophosphoinositol; Gro-3-P, glycerol-3-phosphate; DHAP, dihydroxyacetone phosphate; FA, fatty acids; TAG, triacylglycerol. Steps for which a deletion mutant results in strong or moderate fragmentation deficiency are indicated in red/bold.

Apart from the factors regulating PI(3,5)P_2_, numerous other candidates from the screen are involved in lipid metabolism (Table 5). This suggests that modification of the vacuolar lipid content might play a major role in fragmentation of this organelle. The affected proteins comprise the phospholipases Plb1p, Plb2p and Plb3p, the triacylglycerol lipases Tgl2p and Tgl5p, the putative lipase Lpx1p and Apq12p. Both the acylglycerol lipases and the phospholipases B function in metabolic pathways that converge on diacylglycerol (DAG) and phosphatidic acid (PA) (Fig. 3) [58]. *Saccharomyces cerevisiae* has four phospholipases B, Plb1p, Plb2p, Plb3p and Nte1p, which differ in their substrate specificities. Information on their localization is divergent. Plb1p was detected in the cytoplasm [59] and Plb3p on vacuoles [60]. Other authors assigned all three PLBs to the plasma membrane and the periplasmic space [60]–[63]. Nte1p was found in the endoplasmic reticulum [64].

**Table 5:** Identified mutants in triglyceride metabolism.

ORF	Gene name(s)	Function	Score
YMR008C	PLB1	Phospholipase B	1.6
YMR006C	PLB2	Phospholipase B	1.8
YOL011W	PLB3	Phospholipase B	1.5
YOR081C	TGL5	Triacylglycerol lipase preferring VLCFAs; acyltransferase activity	1.8
YDR058C	TGL2	Acylglycerol lipase	1.5
YIL040W	APQ12	Unknown; mutant accumulates triglycerides	2.0
YOR084W	LPX1	Putative lipase	1.5
YDR503C	LPP1	Lipid phosphate phosphatase	1.0
YDL052C	SLC1	Lyso-PA acyl transferase	1.3
YNR008W	LRO1	Acyl transferase	1.3

Yeast possesses five TAG lipases, which hydrolyze neutral lipids from lipid particles [65]. They prefer substrates with different fatty acyl chain lengths. Tgl5p, which showed a strong fragmentation defect in the screen, mobilizes preferentially long chain fatty acids [66]. Deletion of Tgl2p produced a moderate defect (Table 5) but none of the other TAG lipases, which mobilize small or medium size fatty acids, emerged from our screen. However, we observed a strong phenotype for a mutant in APQ12. The activity of Apq12p is not clear but its deletion leads to a strong accumulation of TAG [67], which could also drain the pools of DAG and PA.

TAGs are major energy stores in the cell. However, in recent years it is increasingly becoming clear that mobilization of TAGs by TAG lipases is intimately linked to growth and development of cells, even under nutrient-rich conditions [68]–[70]. In phases of rapid cell growth, for example, TAG hydrolysis may liberate building blocks for rapid synthesis of the large amount of phospholipids that are needed to synthesize a new daughter cell. As our cells had ample supply of the carbon source glucose it appears unlikely that the TAG-related enzymes might influence vacuole fragmentation as part of an energy-supply mechanism. Given the spatial proximity and association of lipid bodies and vacuoles we favor the alternative hypothesis that the products of TAG hydrolysis might influence vacuole fragmentation by changing the lipid composition of the organelle.

The screen identified multiple enzymes initiating metabolic pathways that converge on DAG and PA. Two possible reasons for this are conceivable: First, rather than DAG and PA, each of the direct products of phospholipases B and the TAG lipases could play a role for fragmentation. Given the diversity of these compounds we find this less likely than the second possibility, that PA and DAG might be the relevant metabolites. DAG and PA induce negative membrane curvature [71], [72], i.e. they convey a tendency to curve membrane leaflets towards their headgroups. They could directly assist in or even drive a deformation of the bilayer and the fission of vesicles [73]. Furthermore, DAG and PA exist in equilibrium with TAGs. TAG metabolism could thus influence vacuolar shape changes via its contribution to synthesis of lipids, e.g. of phosphatidylinositol [74].

Why did we not identify more enzymes for the intermediate steps leading to DAG and PA? Since the screen is based on mutations of single genes, redundancy could prevent the development of strong phenotypes. Indeed, the pathways producing DAG and PA (Fig. 3) are catalyzed by redundant enzymes. Acylation of Gro-3-P can be performed by Gpt2p and Sct1p. It is one of the two pathways to produce lyso-PA and then PA [75]. Alternatively, lyso-PA can be generated from dihydroxyacetone phosphate (DHAP) via Gpt2p, Sct1p and subsequent reduction by Ayr1p [76]. The absence of a significant phenotype for *Δayr1* suggests that production of lyso-PA via the DHAP pathway may be less important for vacuole fragmentation than the Gro-3-P pathway. Further acylation of lyso-PA to PA can be performed by Slc1p or Ale1p [77]. Slc1p was recovered in the screen, but only with a moderate defect. Also alternative pathways exist: PA can originate from the action of phospholipase D and DAG can be produced by PA phosphatase, by phospholipase C and by TAG hydrolysis. The screen revealed no significant defects for mutants in phospholipases C (*Δplc1*) and D (*Δspo14*), suggesting that these pathways may not play a major role for vacuole fragmentation. Of the three proteins with PA phosphatase activity (Pah1p, Lpp1p, and Dpp1p) [78], [79] LPP1 emerged from the screen, but only with a weak phenotype. DAG is acylated to TAG by different enzymes, Lro1p, Are1p, Are2p and Dga1p [80]. Lro1p and Dga1p have the major activity, with Dga1p being most active in stationary phase and Lro1p in the exponential phase [81]. Deletion of Lro1p exhibited a moderate defect in fragmentation activity, while deletion of Are1p showed no effect and deletion of Are2p and Dga1p gave only weak phenotypes. Redundancy might have prevented the observation of stronger phenotypes in all these cases. The situation is further complicated by the fact is that cell biological aspects of the respective enzymes, such as their localization, mode of activation etc. are still incompletely understood. Resorting to an *in vitro* system that reconstitutes fragmentation of isolated vacuoles [27] could help to circumvent some of the problems resulting from redundancy because using the purified organelle might reduce the contribution of enzymes not associated with the vacuolar or pre-vacuolar compartments.

### Components of Endosomal/Vacuolar Protein Traffic

Numerous proteins affecting vacuolar structure and protein trafficking were identified in the screen (Table 3). Among those is Ypt7p, a Rab-GTPase regulating the activation of the HOPS complex. HOPS promotes membrane tethering during vacuolar fusion [82], [83] and the subsequent opening of fusion pores [84]. The HOPS subunit Vps33p also showed a strong fragmentation defect (Table 3). This result is consistent with the hypothesis of an intimate connection and mutual regulation of vacuole fragmentation and fusion [43], which had been proposed based on the fact that the dynamin-like GTPase Vps1p, which is necessary for vacuolar fragmentation, is also involved in the reverse process of vacuole fusion. The identification of Ypt7p and Vps33p in our screen suggests that, in turn, a part of the vacuolar fusion machinery may influence the fragmentation of the organelle.

A striking cluster of mutations concerns class E vps genes, which encode proteins of the pre-vacuolar compartment (PVC), the equivalent of late endosomes in mammalian cells (Table 3) [85]. We identified subunits of the CORVET complex, Vps3p (consistent with earlier observations [42]) and Vps8p, its associated Rab-GTPase Vps21p, the Vps21p-GEF Vps9p, and the endosomal SNARE Pep12p. In addition, we recovered 9 mutations in proteins of the ESCRT machinery, which forms intra-lumenal vesicles of PVCs and sorts proteins into them [86]. Those mutants include the ESCRT-I subunits Mvb12p and Vps28p, the ESCRT-II subunits Snf8p and Vps36p, the ESCRT-III subunit Snf7p, and three interactors of the chaperone Vps4p (Did2p, Vta1p and Vps60p), which dissociates ESCRT-III complexes. Furthermore, Bro1p was recovered, which interacts with ESCRT-III and promotes the removal of ubiquitin from proteins sorted into the lumenal vesicles.

The ESCRT machinery sorts proteins and it deforms and severs membranes during MVB formation, virus budding and cell division [55], [86] [87]. Thus, it has properties that could promote vacuole fragmentation directly. The ESCRT mutants do share, however, the common feature of accumulating the “class E compartment”, a degenerated form of the late endosomal/prevacuolar compartment which concentrates much of the vacuolar H^+^-ATPases that should normally be delivered to the vacuoles [55]. Vacuolar acidification being necessary for fragmentation, future studies will be to dissect whether the ESCRT machinery has a direct role in vacuole fragmentation via its membrane-deforming activity, or whether the mutants affect this process indirectly.

### Fragmentation of Vacuoles is Influenced by Adaptin Mutations

Adaptins form another cluster of mutations affecting vacuole fragmentation, which showed a strong phenotype. They comprise the Apl5p and Aps3p subunits of AP-3 (Table 6), an adaptor complex that mediates protein transport proteins between Golgi and vacuoles [88]. AP-3-dependent transport is regulated by the casein kinase Yck1p [89]–[91], which also emerged from our screen with a strong defect. The other AP-3 subunits Apl6p and Apm3p were not detected. Δ*apl6*, however, results in a weaker transport defect of alkaline phosphatase than the other subunits [91]. We also identified three AP-1 subunits (Apl2p, Apl4p, Aps1p) and Yap180p, a protein promoting the formation of clathrin cages [92] (Table 6). AP-1 is involved in traffic between the TGN and endosomes and Yap180p acts in endocytosis. Interestingly, both Apl4p and Yap180p interact with the Fab1p complex subunit Vac14p [33] and deletion of Apl4p reduces the levels of PI(3,5)P_2_ [93]. Furthermore, overexpression of Fab1p can rescue sorting defects caused by inactivation of AP-1.

**Table 6:** Adaptin- and cytoskeleton-related mutants.

ORF	Gene name(s)	Function	Score	
YKL135C	APL2	β-subunit of AP-1 complex		3.0
YPR029C	APL4	γ-subunit of AP-1 complex		1.8
YLR170C	APS1	σ-subunit of AP-1 complex		1.7
YPL195W	APL5	∂-Subunit of AP-3 complex		1.5
YJL024C	APS3	σ-Subunit of AP-3 complex		2.0
YDR488C	PAC11	Dynein intermediate chain		2.0
YHR161C	YAP1801	Clathrin cage assembly		1.7
YHR135C	YCK1	Septin assembly, endocytosis		1.8

Three simple hypotheses on the involvement of adaptins can be formulated, which are not mutually exclusive: First, sorting of proteins by the clathrin/adaptor protein system to the vacuoles might be necessary to correctly equip the organelle with all necessary fragmentation factors. Second, the effects of AP-1 mutations on vacuole fragmentation might be due to altered PI(3,5)P_2_ levels. This appears likely because Apl4p and Yap180 interact with Vac14p [33] and because *Δapl4* cells show reduced levels of PI(3,5)P_2_ [93]. Furthermore, overexpression of FAB1 can rescue sorting defects caused by inactivation of AP-1. Third, clathrin cages and/or adaptor proteins might bind directly to the surface of the vacuoles to induce or stabilize curvature of the membrane, thereby supporting its fragmentation into small vacuoles. In this respect it is interesting that clathrin forms not only curved coats but also extensive planar lattices, as shown on the plasma membranes of mammalian cells. These forms are inter-convertible [94]–[96]. In mammalian cells, two proteins of the AP-3 complex and clathrin networks were visualized on lysosomes, the functional equivalents of yeast vacuoles [97], [98]. Furthermore, clathrin can also interact with the ESCRT machinery, which is well represented among the mutants identified in our screen [99].

In sum, our screen has uncovered numerous novel factors required for vacuole fragmentation. They provide strong starting points for future targeted analyses exploring their mode action and the mechanism of vacuole fragmentation in detail.

## Materials and Methods

### Reagents

Sources of important chemicals: N-(3-triethylammoniumpropyl)-4-(p-diethylaminophenylhexatrienyl)-pyridinium dibromide (FM4-64, Synaptored TMC2) from Biotium; amino acids from AppliChem; yeast nitrogen base without amino acids from Difco. D+ -raffinose penta-hydrate from Carl Roth. Yeast extract and poly peptone from Pronadisa. 96-well culture plates, 96-well optical bottom plates and non treated coverglass base from Nunc.

### Strains

We used the complete set of *Saccharomyces cerevisiae* non-essential gene deletion strains in haploid BY4741 Mat alpha hisΔ1 met15Δ0 ura3Δ0 [44], which is available from Euroscarf. The collection was used in parallel for another screen in the lab. For the purpose of this experiment the plasmid pRS315-GFP-Nop1 (LEU^+^) had been inserted into all strains by a robotized method in the laboratory of Claudio de Virgilio [100].

### In vivo Vacuole Fragmentation

Yeast was precultured to saturation at 30°C in 96-well plates in HC dropout medium lacking leucine (HC-Leu^-^). From these precultures, new 96-well plates were inoculated in 200 µl HC-Leu^-^. These cultures grew (15 h, 25°C, 150 rpm) in logarithmic phase (OD_600_<1) over night. 20 µl of these cultures were diluted with YPD in to a final volume of 200 µl and were supplemented with 4 µl of FM4-64 from a 1 mM stock in DMSO, yielding final concentration of 20 µM [28]. Cells were incubated (1 h, 27°C, 150 rpm). Plates were then centrifuged 2′ at 3000 rpm at room temperature and the medium was exchanged for YPD, for HC, or for YPD diluted 5× with water. The cells were chased in these media for 2 hours at 27°C and 150 rpm. 50 µl of culture were then transferred into optical 96-wells plates for microscopy. Cells were analyzed on an inverted Zeiss Axioplan fluorescence microscope (equipped with a 100×/1.4 NA lens) before the reaction. Then, 0.4 M NaCl were added from a 5 M stock and mixed by pipetting up and down three times. The cells were left at RT (25°C) for 10 min and analyzed again.

### Scoring

We attributed a score S to each mutant according to the loss of fragmentation activity; S = 1 for 5–10% of cells with non-fragmented vacuoles, S = 2 for 20–50% of cells with non-fragmented vacuoles, S = 3 for 60–90% and S = 4 in the case of all cells with completely non-fragmented vacuoles. In order to integrate the results from the three screening approaches we calculated a parameter reflecting the level of deficiency in fragmentation activity. Some mutants did not grow under all conditions. In order to take this into account, we defined an average phenotypic score P by summing up the scores S_i_ from all rounds of screening in which the mutant could be analyzed and divided this sum by the number of rounds n. P can thus take values from 0 to 4. Mutant phenotypes were classified into three categories: Small defects of fragmentation with 0<P<1.3, moderate defects with 1.3≤P<1.6 as, strong defects with 1.6≤P<2 and very strong defects with 2≤P≤4.

## Supporting Information

Table S1 **List of primary screening results.** Screens were performed in three media, HC, YPD and diluted YPD. Diluted YPD gives the strongest fragmentation response and the easiest readout. Therefore, this screen was performed twice. Clones or plates showing irregularities, such as contaminations or atypical vacuole structure or cell morphology, were re-analyzed separately.
(XLSX)
